# A Remarkable Sensitive

**Published:** 1889-02

**Authors:** 


### A REMARKABLE SENSITIVE.

Miss Lulu Billings, the only daughter of Mr. E. G. Billings, of Rochester, is creating great excitement by her remarkable spiritual manifestations. Miss Billings is a tall, slim brunette, about twenty-nine years of age, with a rather pretty face and quiet, attractive manner. It seems she is endowed with a supernatural musical power. For several years Miss Lulu has been able to sing while in a trance state, but her parents have been so averse to having the public know the fact that until recently very few have listened to the fair musician during one of her "spirit" performances.

The young lady takes her seat at the piano, and, after a few nervous movements of her head, passes into an unconscious, or, as she says, "trance state," during which she plays and sings with great ease and skill.

She has no knowledge of music whatever, save what her mother has taught her upon the piano, and even here her skill is by no means above the average of many girls of eighteen years of age, yet her playing while entranced is beautiful in the extreme. She also plays in a masterly manner while under the power of the "spirits" upon the guitar, flute, cornet, violin and harp, although she has never received instruction on any of them.

She says she is controlled and directed by an Italian musician and scholar who has been dead several centuries, and who, she says, is named Ingrelio. Under his direction she improvises rare harmonies, but her playing while not in the trance state is of a very mediocre order. She has a sweet soprano voice of considerable range.

According to Mrs. Billings, about nine years ago the family first learned that Lulu was controlled by spirits. One night they were calling upon some neighbors who were spiritualists, when Lulu went into the sitting-room and began playing upon the piano. The room was entirely dark. They heard her playing familiar songs, but after a time the music was of such an order and so strange that they went in and lighted the gas to see who was playing. She says that her daughter sat there with her hair hanging over her eyes playing like mad. She ran to the piano and shook Lulu, who gave a scream and, fainting, fell to the floor. From that time she has continued to develop her strange power. Mrs. Billings says that when Emma Abbott was stopping here she sat in the window of the hotel and listened in amazement to Lulu's singing. She made inquiries regarding her, and accompanied by members of her company called to hear her sing and pronounced her voice superb.

Mrs. Billings says her daughter sings in Spanish, French, Italian, German, Chinese and in the ancient Hindoo dialect. Mr. Billings does not believe in spirits despite the wonderful performances of his daughter, but says he cannot account for her power.

